# Partial laparoscopic nephrectomy: what really matters?

**DOI:** 10.1590/S1677-5538.IBJU.2020.0167.1

**Published:** 2020-11-18

**Authors:** Leonardo Oliveira Reis, Cristiano Trindade de Andrade

**Affiliations:** 1 Universidade Estadual de Campinas UroScience CampinasSP Brasil UroScience, Universidade Estadual de Campinas - Unicamp, Campinas, SP, Brasil; 2 Pontifícia Universidade Católica de Campinas PUC-Campinas CampinasSP Brasil Pontifícia Universidade Católica de Campinas, PUC-Campinas, Campinas, SP, Brasil

## COMMENT

Partial nephrectomy has been proven efficient in most situations in which residual functional parenchyma can be safely preserved (1). Several factors can influence the laparoscopic partial nephrectomy outcome. Preoperative renal function, ischemia type, ischemia duration, and the remnant parenchyma (2). The ischemia time seems to be one of the most relevant factors, although to perform an off-clamp partial nephrectomy can be challenging.

The best ischemia type and duration are still controversial. A similar decrease in glomerular filtration rate after three months of follow-up was observed when compared cold and warm ischemia, although the cold ischemia time was significantly longer (45 min vs 22 min) (3). An off-clamp approach is an attractive option in favorable tumors, once the ischemia and reperfusion injury persists beyond the clamping period. The off-clamp surgery shows benefits in the renal function recovery, especially in solitary kidneys in the short-term (4–6).

The off-clamp procedure is associated with a faster renal function recovery, but it is necessary to be prepared to change the surgical plan, especially during bleeding. Finally, the surgeon's experience continues to be essential to choose the best approach for each procedure.

The transperitoneal or retroperitoneal laparoscopic approach also have their pros and cons. The transperitoneal access has the advantage of larger working space and natural orientation to the natural landmarks. The retroperitoneal has a decreased risk of intraperitoneal structures damage and direct access to the renal hilum (7). Therefore, the retroperitoneal approach may offer modest benefits for operative time and have utility in posterior tumors (8).

Laparoscopic partial nephrectomy is technically challenging, and to have success in such minimally invasive surgery, a meticulous preoperative evaluation is mandatory, including kidney anatomy, vasculature, and tumor features. Imaging of renal anatomy and vasculature is essential for surgical planning, especially associated with nephrometry scoring systems (2).

While there are valuable tools to predict surgical challenges and to decrease difficulties during the procedure, there are still features to be evaluated such as the adherent perinephric fat, a non–tumor-related factor that can complicate the surgery by limiting kidney mobilization and tumor isolation, increasing the operative time (9).

Recently, in the International Brazilian Journal of Urology, Mercimek and Ozden have explored the functional impact of surgeon option of trans- or retroperitoneal access in a retrospective off-clamp laparoscopic partial nephrectomy series (10), with similar above 90% Pentafecta outcomes, which consists in warm ischemia time ≤25 min, negative surgical margins, no perioperative complications, renal function expressed as over 90% glomerular filtration rate (GFR) preservation, and no upgrade of chronic kidney disease (CKD) stage at postoperative 12 months (11).

Although the reported significantly higher ΔeGFR (mL/min/1.73m2) in the transperitoneal access (10) should be analyzed with care and further explored in future studies, >90% of baseline function maintenance was the rule as expected, considering the off-clamp tactic (12). On the other hand, data is limited to serum creatinine levels and subject to variations on estimated glomerular filtration rate (eGFR) in a low volume surgeon scenario (13).

Though a tiny room for improvement considering the high Pentafecta rates, a bigger and prospective randomized trial is necessary to mitigate confounders related to the renal vascular anatomy, R.E.N.A.L nephrometry score (RNS), tumor characteristics, and vascular supplies of the tumor that have dictated the surgeon's preference on access technique (10).
